# Parity affects mammary development in late-pregnant swine

**DOI:** 10.1093/tas/txae037

**Published:** 2024-03-23

**Authors:** Chantal Farmer, Jakob C Johannsen, Caroline Gillies, Lee-Anne Huber, Russell C Hovey

**Affiliations:** Agriculture and Agri-Food Canada, Sherbrooke Research and Development Centre, 2000 College, Sherbrooke, QC, CanadaJ1M 0C8; Department of Animal and Veterinary Sciences, Aarhus University, DK-8830 Tjele, Denmark; Department of Animal Biosciences, University of Guelph, Guelph, ON, CanadaN1G 2W1; Department of Animal Biosciences, University of Guelph, Guelph, ON, CanadaN1G 2W1; Department of Animal Science, University of California, Davis, CA 95616, USA

**Keywords:** gestation, gilt, mammary development, parity, sow

## Abstract

The goal of this project was to determine whether various measures of mammary development differed between gilts and multiparous sows at the end of gestation. During gestation, Yorkshire × Landrace gilts (*n* = 19) and sows (second and third gestations, *n* = 17) were fed one daily meal of a conventional corn-based diet, where the amount fed was based on body weight (BW) and backfat thickness (BF) at mating. On day 110 ± 1 of gestation, a jugular blood sample was obtained from all gilts and sows to measure insulin-like growth factor-1 (IGF-1), glucose, free fatty acids, and urea. On that same day, BW and BF were measured and animals were euthanized. Mammary glands from one side of the udder were dissected for compositional analyses. The fifth gland of the contralateral row of mammary glands was sampled for histology and immunohistochemical localization of Ki67. There was less total parenchyma (1,437.4 vs. 2,004.7 ± 127.1 g; *P* < 0.001) and total extraparenchymal tissue (1,691.0 vs. 2,407.0 ± 125.3 g; *P* < 0.001) in mammary glands of gilts compared to those from sows. When these values were expressed per kg BW (226.0 and 284.0 ± 2.7 kg for gilts and sows, respectively), parenchymal mass did not differ (*P* > 0.10), while extraparenchymal tissue weight tended to be less in gilts than sows (*P* = 0.07). All components within the parenchyma differed by parity (*P* < 0.001). Specifically, parenchymal tissue from gilts contained a greater proportion of fat and dry matter (DM), a lower proportion of protein, and lower concentrations of DNA (6.59 vs. 9.35 ± 0.53 mg/g DM) and RNA (7.76 vs. 12.33 ± 0.70 mg/g DM) than that from sows. On the other hand, the circumference of alveolar lumens was greater in gilts than sows (*P* < 0.001), while the percentage of epithelial cells that were positive for Ki67, a marker of cell proliferation, was greater in sows than gilts (*P <* 0.05). Circulating concentrations of IGF-1 were greater in gilts than in multiparous sows (45.0 vs. 27.3 ± 2.8 ng/mL, *P* < 0.001). None of the other blood variables were changed by parity. Results show a marked effect of parity on mammary gland development in swine. At the end of gestation, the mammary glands of gilts had less parenchyma with lower epithelial proliferation than glands from multiparous sows. These differences could alter the response of mammary tissue to various nutritional or endocrine signals. This information is crucial for the development of management strategies designed to maximize sow milk yield.

## Teaser

Parity influences mammary development of pigs in late gestation, with gilts having less developed mammary parenchyma than multiparous sows. Such a difference will likely affect the response of mammary tissue to nutritional or other treatments during gestation.

## Introduction

The extent of mammary development that occurs before the onset of lactation is a major determinant of milk production in swine (Head and Williams, 1991). Maximizing mammary development during late gestation is therefore crucial for the optimal growth of suckling piglets, and this is even more important with the current large litter sizes. Nutrition can affect the growth of mammary tissue in pregnant swine and many feeding strategies have been assessed to maximize this development. Feeding different protein and energy levels during gestation in order to alter body condition showed that obese gilts (36 mm backfat) have poorer mammary development than leaner gilts (24 mm backfat), with a drastic reduction in mammary DNA concentration (Head and Williams, 1991). It was later demonstrated that in order to optimize mammary development, the feed intake of gestating gilts should be adjusted to achieve a backfat thickness between 16 and 26 mm before farrowing (Farmer et al., 2016). Increasing energy intake from 5.76 to 10.5 Mcal ME/d between days 75 and 105 of gestation decreased the weight of milk-synthesizing parenchyma by the end of the treatment (Weldon et al., 1991). Even though increasing crude protein intake from 216 to 330 g/d during that same period did not alter mammary development (Weldon et al., 1991), a 40% increase in dietary standard ileal digestible (SID) lysine content, via the inclusion of additional soybean meal, from days 90 to 110 of gestation increased the weight of mammary parenchyma in gilts by 44% (Farmer et al., 2022). Increasing circulating concentrations of insulin-like growth factor-1 (IGF-1) in late gestation also brought about a 22% increase in mammary parenchymal tissue mass (Farmer and Langendijk, 2019). It is important to note that most studies on mammary development in pregnant swine have been performed with gilts (Head and Williams, 1991; Weldon et al., 1991; Sorensen et al., 2002; Ji et al. 2006; Farmer et al., 2016, 2022; Farmer and Langendijk, 2019), with few using multiparous sows (Farmer et al., 2023) that represent a large portion of the herd in commercial operations.

Mammary glands undergo drastic quantitative (Sorensen et al., 2002) and histological (Ji et al., 2006) changes during the first gestation. Further mammary growth occurs throughout lactation (Kim et al., 1999), followed by involution after weaning (Ford et al., 2003). These last authors noted that at the end of the involution process, glands that were suckled during lactation were larger than non-suckled glands, raising the prospect that mammary glands that were suckled in first lactation may show enhanced development before the onset of the subsequent lactation. Hence, parity may affect mammary gland development in late-pregnant swine, yet this was never investigated. What was recently demonstrated, however, is that parity affects the response of mammary tissue to a nutritional treatment during late gestation. Indeed, when the same dietary treatment was applied to either gilts or multiparous sows, it had different impacts on mammogenesis. Increasing SID lysine content by 40%, via the inclusion of additional soybean meal, from days 90 to 110 of gestation increased mammary parenchymal mass in gilts (Farmer et al., 2022), but had no effect on mammary development in sows during their second and third gestations (Farmer et al., 2023). Those findings suggest that parity may affect the nutritional requirements for mammary tissue in late gestation. In order to design feeding strategies that are best adapted to pregnant swine, it is imperative to understand changes in their mammary gland due to parity. In the present study we therefore sought to determine if various measures of mammary development at the end of gestation differ between gilts and multiparous sows, with the hypothesis that these would be greater in sows that underwent at least one previous lactation compared with gilts.

## Materials and Methods

Animals were cared for according to a recommended code of practice (CCAC, 2009) following procedures approved by the institutional animal care committee of the Sherbrooke Research and Development Center of Agriculture and Agri-Food Canada. These data were previously published in part by Farmer et al. (2022, 2023).

### Animals and Treatments

Thirty-six Yorkshire FAST × Landrace FAST (Groupe Cérès Inc., Saint-Nicolas, QC, Canada) gilts (*n* = 19) or multiparous sows (second gestation, *n* = 9; third gestation, *n* = 8) were bred via artificial insemination using pools of semen from Duroc Super Gain Plus boars (Center d’Insémination Porcine du Québec, Saint-Lambert-de-Lauzon, QC, Canada). During gestation, gilts and sows were housed in individual pens (1.5 × 2.4 m) and were fed one daily meal (0800 hours) of a conventional corn- and soybean meal-based diet (12.75 MJ/kg DE, 11.24% CP, 0.57% SID lysine for gilts, and 11.68 MJ/kg DE, 13.99% CP, 0.52% SID lysine for multiparous sows) that met the estimated nutrient and energy requirements for gilts and sows, respectively. The amounts fed from mating to day 89 of gestation were determined according to industry specifications based on body weight (BW) and backfat thickness (BF), which amounted to gilts and sows being fed 2.65 and 2.60 kg/d, respectively, on day 90 of gestation. At the time of mating and on days 90 and 110 of gestation, animals were weighed and their BF was measured ultrasonically (WED-3000, Schenzhen Well D Medical Electronics Co., Guangdong, China) at P2 of the last rib. On day 110 ± 1 of gestation, blood samples were collected by jugular venipuncture before the meal (between 0700 and 0800 hours) following a 16 h fast. Gilts and sows were necropsied on the same day to obtain mammary glands for compositional analyses, histology, and immunohistochemistry measures. The uterus was removed and fetuses were counted and weighed, while the ovaries were weighed and the number of corpora lutea counted.

### Blood Collection and Assays

The circulating concentrations of IGF-1, glucose, free fatty acids (FFA), and urea were measured. Blood samples for urea (20 mL) were collected into vacutainer tubes without anticoagulant (Becton Dickinson, Franklin Lakes, NJ) and held at room temperature for 3 h, stored overnight at 4 °C, centrifuged for 12 min at 1,800 × *g* at 4 °C the following day, before the serum was harvested. Blood samples for IGF-1 and FFA assays (30 mL) were collected into EDTA tubes (Becton Dickinson), chilled, and centrifuged within 20 min for 12 min at 1,800 × *g* at 4 °C, from which plasma was immediately recovered. Lastly, blood for glucose analysis (6 mL) was collected into potassium oxalate/sodium fluoride tubes, held on ice, and centrifuged within 20 min at 1,800 × *g* for 12 min at 4 °C, and the plasma recovered. Serum and plasma samples were stored at −20 °C. Concentrations of IGF-1 were measured with a commercial RIA kit for human IGF-1 (ALPCO Diagnostics, Salem, NH) with minor modifications as detailed previously (Plante et al., 2011). The assay was validated using a pooled sample of plasma from sows as described (Plante et al., 2011). The sensitivity of the assay was 0.10 ng/mL, while the intra- and interassay CVs were 5.23% and 10.79%, respectively. Glucose was measured by an enzymatic colorimetric method (Wako Chemicals, Richmond, VA). Intra- and interassay CVs were 2.37% and 5.32%, respectively. Urea was measured colorimetrically using an autoanalyzer (Auto-Analyzer 3; Technicon Instruments Inc., Tarrytown, NY) according to the method of Huntington (1984). Intra- and interassay CVs were 1.83% and 0.96%, respectively. Concentrations of FFA were measured by colorimetry (Wako Chemicals) having intra- and interassay CVs of 1.43% and 3.49%, respectively.

### Mammary Gland Measurements

At necropsy, all mammary glands from one side of the udder (averaging 7.68 ± 0.13 and 7.77 ± 0.14 teats for gilts and sows, respectively) were excised for measures of mammary composition. Glands were frozen and stored at −20 °C, then were cut transversally into 2 cm slices while frozen, prior to being stored again at −20 °C. Each slice was later trimmed of skin and teats and the parenchyma dissected from the surrounding extraparenchymal tissue at 4 °C. Mammary parenchyma was defined as the pinkish tissue that contains duct and alveolar cells, and also some interspersed stromal cells, whereas the white extraparenchymal tissue consisted of adipose tissue. Parenchyma from all glands was homogenized together, and a representative sample was used for compositional analysis. The RNA content was measured by ultraviolet spectrophotometry (Volkin and Cohn, 1954), and the DNA content was measured by using a fluorescence-based assay (Labarca and Paigen, 1980). Dry matter (DM; Method 950.46; AOAC, 2005), protein (Method 928.08; AOAC, 2005), and lipid contents (Method 991.36, AOAC, 2005) of parenchyma were also measured. Both RNA and DNA contents were reported on a DM basis.

The contralateral row of mammary glands was used for histology and immunohistochemistry. Samples from the fifth gland within the mammary row were collected, fixed in 4% neutral-buffered paraformaldehyde for 24 h at 4 °C, then washed twice with and stored in 70% ethanol prior to embedding in paraffin.

### Histology and Immunohistochemistry

Paraffin-embedded samples from 32 females (*n* = 16 gilts and *n* = 16 sows) were sectioned at 4.5 μm and mounted on charged slides. Epithelial cell proliferation was determined from immunohistochemical localization of Ki67 in sections that were rehydrated and pretreated with 0.3% Triton-X in PBS. Antigen retrieval was performed by steaming in Tris (10 mM) EDTA (1 mM) buffer (pH 9) before blocking with 10% horse serum for 1 h at room temperature. Sections were then incubated with a biotinylated rat monoclonal anti-Ki67 antibody (RRID:AB_2572794; 1:200; Thermo Fisher Scientific, Waltham, MA) and a mouse monoclonal antibody against β-catenin (RRID:AB_1030943; 1:200: Cell Signaling, Danvers, MA) in 10% horse serum overnight at 4 °C. Sections were then rinsed in 0.05% PBS-Tween 20 and incubated in 10% horse serum overnight at 4 °C with both an Alexa Fluor 647 streptavidin-conjugated donkey anti-rat secondary antibody (RRID:AB_2340694; 1:500) to localize Ki67, and an Alexa Fluor 488-conjugated anti-mouse secondary antibody (RRID:AB_2340694; 1:200) to localize epithelium-specific β-catenin. Sections were counterstained with DAPI (1:1,000) before coverslipping. Randomly selected fields in the four quadrants of each section were imaged using a 20× objective on an Olympus BX51 microscope fitted with a cooled CCD digital camera (QICAM; Q Imaging) driven by Q Capture Pro 7 software (Q Imaging). Monochrome images for each wavelength were captured using the same settings and exposures across all specimens. The incidence of Ki67-positive nuclei among the β-catenin-positive epithelium in each field was quantified using an in-house macro in FIJI (https://imagej.net/software/fiji/) utilizing the StarDist plugin. The Ki67-positive nuclei were tagged manually, and their incidence was expressed relative to the corresponding number of DAPI-positive epithelial nuclei to determine the percent labeling index. All epithelial cells within a given field (range from 330 to 1,083 nuclei) were analyzed. The alveolar circumference was determined by manually tracing the apical membrane of all complete alveoli within a composite image using FIJI, as calibrated against a stage micrometer. The average alveolar perimeter was determined from an average of 108 alveoli analyzed per female.

### Statistical Analyses

The MIXED procedure of SAS (SAS Inst. Inc., Cary, NC) was used for statistical analyses. A univariate model was used for all measured variables and included the effect of treatment (parity), with the residual error being the error term used to test for main effects of treatment. An ANOVA with heterogeneous variances was used when necessary. Data in tables are presented as least squares means ± maximal SEM.

## Results

### Growth, Ovarian, Fetal, and Blood Variables

The mean BW of gilts and multiparous sows on day 110 of gestation was 226.0 and 284.0 ± 2.7 kg, respectively (*P* < 0.001), with corresponding average BF of 17.6 and 19.5 ± 0.9 mm, respectively (*P *> 0.10). The total number of corpora lutea from both ovaries was greater in multiparous sows than gilts (27.5 vs. 20.5 ± 1.1, *P* < 0.001), as was the combined weight of the ovaries (37.2 vs. 24.0 ± 1.05 g, *P* < 0.001). There was a tendency for there to be more fetuses in multiparous sows than gilts (16.7 vs. 14.3 ± 0.9, respectively, *P* = 0.08), while the BW of these fetuses was not different across parities (1.23 vs. 1.27 ± 0.04 kg for gilts and multiparous sows, respectively; *P* > 0.10).

Circulating concentrations of IGF-1, glucose, FFA, and urea on day 110 of gestation are shown in Table 1. There were no differences in glucose, FFA, or urea concentrations across parities, whereas the average concentration of IGF-1 was greater (*P* < 0.001) in gilts than in multiparous sows.

**Table 1.** Circulating concentrations of IGF-1, glucose, free fatty acids, and urea from gilts (*n* = 19) or multiparous sows (*n* = 17) on day 110 of gestation

	Treatment			
Variable measured	Gilts	Multiparous sows	SEM^1^	*P*-value
IGF-1, ng/mL	45.0	27.2	2.8	<0.001
Glucose, mMol/L	3.32	3.34	0.07	0.80
FFA, µEq/L	144.1	214.1	30.5	0.10
Urea, mMol/L	6.44	6.68	0.24	0.47

^1^Maximum value for the standard error of the mean (SEM).

### Mammary Gland Variables

**Figure 1.**
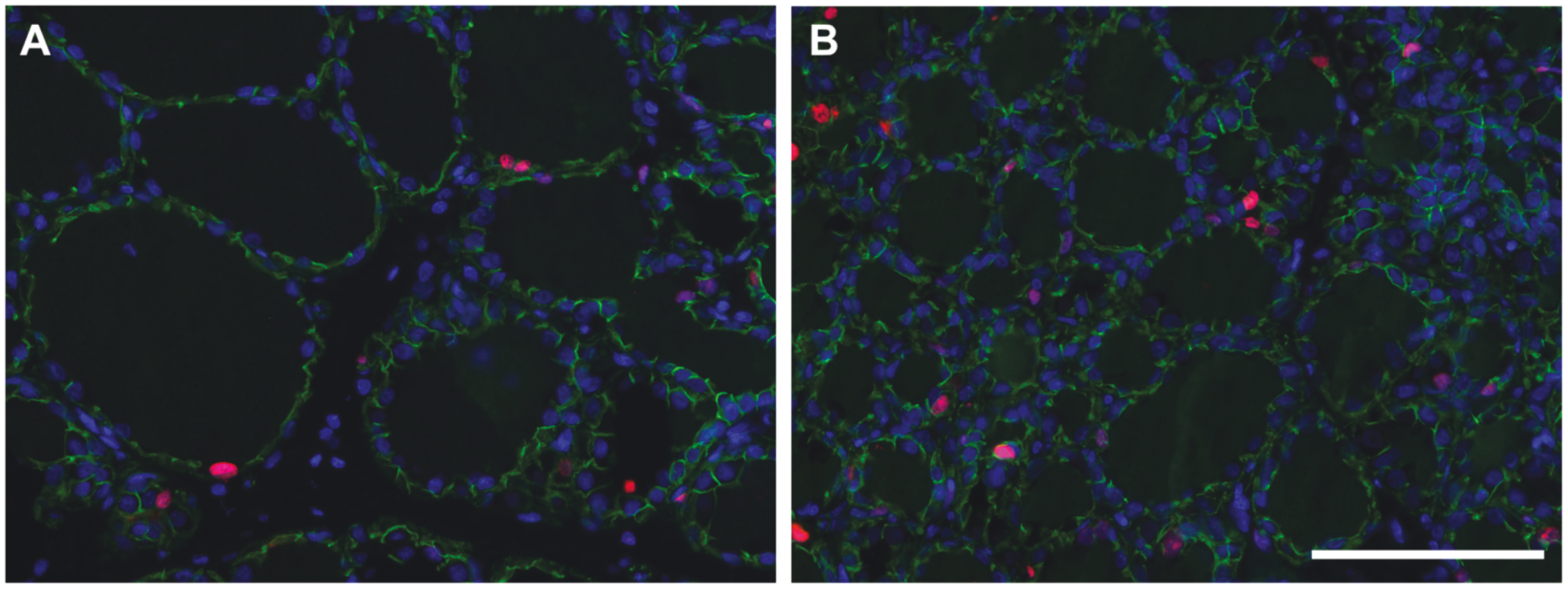
Fluorescent immunohistochemistry for β-catenin (cellular membranes, green) and Ki67 (nuclei, red), counterstained with DAPI (nuclei, blue), on mammary tissue from a gilt (A) and sow (B). The Ki67 labeling indices for panels A and B were 3.6% and 4.9%, respectively. Scale bar = 100μm.

Mammary gland composition on day 110 of gestation is shown in Table 2. There was less total parenchyma and total extraparenchymal tissue (*P* < 0.001) in mammary glands of gilts compared with multiparous sows. However, when these values were expressed per kilogram BW, there was no difference across the parity group for parenchymal content, and only a tendency for the weight of extraparenchymal tissue to be less in gilts (*P* = 0.07). Parity altered all components of the parenchyma (*P* < 0.001). Specifically, parenchyma from gilts had greater fat and DM percentages, lower protein percent, and lower concentrations of DNA and RNA than that from multiparous sows. The total amount of parenchymal fat was similar across parities, whereas total protein (*P* < 0.01), total DNA, and total RNA (*P* ≤ 0.001) in parenchyma was lower in gilts compared with multiparous sows. When these parenchymal components were normalized for BW, there was more total fat (*P* < 0.001) and less total RNA (*P *< 0.01) in gilts than multiparous sows, while there was no effect of parity on total protein or DNA content. However, both total DNA per gland (*P* < 0.01) and total RNA per gland (*P* < 0.001) were less in gilts than multiparous sows. The circumference of alveolar lumens was greater in gilts than multiparous sows (*P* < 0.001, Figure 1), and the percentage of epithelial cells that were positive for Ki67, an indicator of cell proliferation, was greater in sows (*P *< 0.05, Figure 1).

**Table 2.** Mammary gland composition (*n* = 19 for gilts, *n* = 17 for sows) and immunohistochemical variables (*n* = 16 for gilts, *n* = 16 for sows) for parenchymal tissue from gilts or multiparous sows on day 110 of gestation

	Treatment			
Variable measured	Gilts	Multiparous sows	SEM^1^	*P-*value
Extraparenchymal tissue, g	1,691.0	2,407.0	125.3	<0.001
Extraparenchymal tissue/BW, g/kg	0.75	0.85	0.04	0.07
Parenchymal tissue, g	1,437.4	2,004.7	127.1	<0.001
Parenchymal tissue/BW, g/kg	0.64	0.71	0.04	0.22
Parenchyma/gland^2^, g	189.0	258.7	16.2	0.002
Parenchymal tissue composition				
Dry matter, %	37.8	29.0	1.0	<0.001
Fat, % of dry matter	63.3	49.4	2.1	<0.001
Fat, g total	337.2	289.8	28.3	0.14
Total fat/BW, g/kg	149.4	101.6	9.3	<0.001
Protein, % of dry matter	32.9	44.6	2.1	<0.001
Protein, g total	178.4	256.5	22.3	0.004
Total protein/BW, g/kg	78.9	90.0	7.3	0.23
DNA, mg/g on dry matter basis	6.59	9.35	0.54	<0.001
DNA, g total	3.58	5.28	0.39	0.001
Total DNA/BW, g/kg	1.59	1.86	0.13	0.14
DNA/gland^2^, g	0.47	0.68	0.05	0.002
RNA, mg/g on dry matter basis	7.76	12.33	0.70	<0.001
RNA, g total	4.20	6.97	0.52	<0.001
Total RNA/BW, g/kg	1.86	2.45	0.17	0.009
RNA/gland^2^, g	0.55	0.90	0.07	<0.001
Alveolar circumference, µm	141	95	6	<0.001
Ki67-positive epithelial cells, %	3.51	4.56	0.35	0.04

^1^Maximum value for the standard error of the mean (SEM).

^2^Total value divided by the number of mammary glands from the side of the udder used.

## Discussion

In the present study, we provide the first demonstration that parity affects mammary development in late-pregnant swine. These findings likely impact nutritional recommendations for gestating gilts and sows. Considering that the number of epithelial cells in the mammary glands at the onset of lactation has a major impact on sow milk yield (Head and Williams, 1991), the lower weight of mammary parenchyma and lower index of epithelial cell proliferation in gilts compared with multiparous sows on day 110 of gestation in the current study concurs with the greater milk yield of multiparous vs. primiparous sows (King, 2000). There were similar increases for both extraparenchymal tissue (42.3% greater) and parenchymal tissue (39.5% greater) in sows vs. gilts, although once these were corrected for BW, only extraparenchymal tissue mass tended to be greater. The fact that parenchymal weight was similar after correction for BW underscores the impact of growth still occurring during gestation (hence lower BW) on total mammary tissue in gilts on day 110 of gestation.

The composition of mammary parenchyma also reflected lesser development in gilts compared with multiparous sows, with greater percent fat, lower percent protein, and lower concentrations of DNA and RNA in gilts. Lower total parenchymal DNA and RNA in gilts also indicated less development compared with sows. When these total parenchymal components were expressed per kilogram of BW, differences across parity were no longer apparent for protein and DNA concentrations, while multiparous sows still had a greater concentration of RNA than gilts. This finding of a greater normalized concentration of parenchymal RNA in multiparous sows compared with gilts is novel and suggests that the metabolic activity of mammary epithelial cells, rather than number of cells, could direct the lactational capacity of the glands after the first parity. On the other hand, multiparous sows had a greater index of epithelial cell proliferation and, when considering that they had greater parenchymal concentrations of protein, DNA and RNA, but smaller alveolar circumference, they likely had more alveolar cells per glandular volume. This increase in alveolar cells could lead to greater milk synthesis capacity that would be brought about by the regrowth of this mammary tissue following a first lactation. Indeed, Manjarin et al. (2011) recorded an increase in mammary DNA during lactation in primiparous sows, but not in multiparous sows. In saying this, one consideration is that the proliferation of mammary epithelial cells in gilts was greater on day 90 than on day 110 of gestation (VanKlompenberg et al., 2013), where it is not clear if this is also the case in multiparous sows. Hence, the timing of this measure in the current study may have affected the outcome. Regardless, these proliferation values represent the situation just prior to the onset of lactation in both gilts and sows.

The improved mammary development in multiparous sows reflects the retention of established tissue from the previous lactation. After weaning, mammary glands involute rapidly within seven days, as evidenced by the rapid regression of parenchymal weight and DNA (Ford et al., 2003). These authors noted that suckled mammary glands were larger at the end of lactation than non-suckled glands, and suggested a beneficial effect of teat use on redevelopment, and possibly milk yield, in the next lactation. Such an impact would be due to the fact that glands that are suckled during lactation approximately double in size, hence starting the postweaning involution process at a larger size. Farmer et al. (2012) later demonstrated that a gland suckled in first parity produces more milk in second lactation, alongside enhanced development (i.e., increased parenchymal mass and parenchymal DNA and RNA contents per gland) before weaning. The suckling history of individual glands from sows in the current study is not known, although considering the average litter sizes we expect that almost all glands were previously used.

The lower epithelial proliferation in mammary parenchyma from gilts compared with multiparous sows indicates that mammary cell proliferation is not related to the response to growth-altering treatments during gestation. Indeed, when dietary SID lysine was increased by 40% from days 90 to 110 of gestation in either gilts (Farmer et al., 2022) or multiparous sows (Farmer et al., 2023), only gilts showed a 44% increase in parenchymal mass when fed additional lysine compared with controls (Farmer et al., 2022, 2023). Interestingly, the weight of parenchymal tissue in gilts receiving additional lysine (2,073.6 g) in the former study was similar to that of both control (2,004.7 g) and treated (2,099.3 g) multiparous sows in the subsequent study, which may reflect a threshold above which no further increase is possible. To investigate that hypothesis, it would be interesting to combine a 40% increase in SID lysine intake by late-pregnant gilts with another treatment also known to stimulate mammary development, such as increased concentrations of IGF-1 (Farmer and Langendijk, 2019).

The greater circulating concentration of IGF-1 in gilts in this study can be linked to the fact that, contrary to multiparous sows, gilts are still growing during their first pregnancy. The body growth of gilts seems more responsive to a dietary treatment during gestation than that for sows. Indeed, providing 40% additional dietary SID lysine to gilts from days 90 to 110 of gestation increased their BW gain during the treatment period (28.0 vs. 24.2 ± 0.7 kg; Farmer et al., 2022), whereas no increase occurred in multiparous sows subjected to the same treatment (18.1 vs. 16.6 ± 0.8 kg; Farmer et al., 2023). It is possible that the greater circulating concentrations of IGF-1 in gilts compared with multiparous sows in late gestation did not affect the amount or composition of their mammary parenchyma. Indeed, while it was previously shown that increasing IGF-1 concentrations during late pregnancy can stimulate mammary development of gilts both in terms of parenchymal mass and composition, IGF-1 concentrations of approximately 200 ng/mL were needed to promote a beneficial effect (Farmer and Langendijk, 2019), which is well above concentrations in gilts from the current study. On the other hand, the potential impact of IGF-1 on alveolar expansion is not known since it was not measured in that previous study.

In conclusion, mammary development in late gestation is affected by parity both in terms of the amount and composition of parenchyma. Gilts have less parenchymal tissue containing less protein, DNA and RNA but with greater alveolar expansion than multiparous sows, while sows show greater proliferation of mammary epithelial cells. These differences reflect the greater milk yield of multiparous sows and highlight that responses to various nutritional or other treatments during gestation may differ between the first and subsequent parities.

## References

[CIT0001] AOAC. 2005. Official methods of analysis international. 18th ed. Arlington (VA): Association of the Official Analytical Chemists.

[CIT0002] CCAC. 2009. Canadian Council on Animal Care Guidelines on: the Care and Use of Farm Animals in Research, Teaching and Testing. Ottawa (ON): Canadian Council on Animal Care in Science.

[CIT0003] Farmer, C., C. R. A. Duarte, M. Vignola, and M. F. Palin. 2016. Body condition of gilts at the end of gestation affects their mammary development. J. Anim. Sci. 94:1897–1905. doi: 10.2527/jas.2016-0336 27285687

[CIT0004] Farmer, C., C. Gillies, J. C. Johannsen, R. C. Hovey, and L. A. Huber. 2023. Dietary supplementation with lysine (protein) in late pregnancy does not enhance mammary development in multiparous sows. J. Anim. Sci. 101:1–8. doi: 10.1093/jas/skad385 PMC10746349 37971408

[CIT0005] Farmer, C., and P. Langendijk. 2019. Exogenous porcine somatotropin stimulates mammary development in late-pregnant gilts. J. Anim. Sci. 97:2433–2440. doi: 10.1093/jas/skz136 31066897 PMC6541820

[CIT0006] Farmer, C., M. F. Palin, R. C. Hovey, T. D. Falt, and L. A. Huber. 2022. Dietary supplementation with lysine stimulates mammary development in late-pregnant gilts. J. Anim. Sci. 100:1–11. doi: 10.1093/jas/skac051 PMC9109004 35184195

[CIT0007] Farmer, C., M. F. Palin, P. K. Theil, M. T. Sorensen, and N. Devillers. 2012. Milk production in sows from a teat in second parity is influenced by whether it was suckled in first parity. J. Anim. Sci. 90:3743–3751. doi: 10.2527/jas.2012-5127 22665676

[CIT0008] Ford, J. A. Jr, S. W. Kim, S. L. Rodriguez-Zas, and W. L. Hurley. 2003. Quantification of mammary gland tissue size and composition changes after weaning in sows. J. Anim. Sci. 81:2583–2589. doi: 10.2527/2003.81102583x 14552387

[CIT0009] Head, R. H, and I. H. Williams. 1991. Mammogenesis is influenced by pregnancy nutrition. In: Manipulating pig production III. Werribee (VIC): Australasian Pig Science Association. p. 33.

[CIT0010] Huntington, G. B. 1984. Net absorption of glucose and nitrogenous compounds by lactating holstein cows. J. Dairy Sci. 67:1919–1927. doi: 10.3168/jds.S0022-0302(84)81525-8 6491011

[CIT0011] Ji, F., W. L. Hurley, and S. W. Kim. 2006. Characterization of mammary gland development in pregnant gilts. J. Anim. Sci. 84:579–587. doi: 10.2527/2006.843579x 16478949

[CIT0012] Kim, S. W., W. L. Hurley, I. K. Han, and R. A. Easter. 1999. Changes in tissue composition associated with mammary gland growth during lactation in sows. J. Anim. Sci. 77:2510–2516. doi: 10.2527/1999.7792510x 10492459

[CIT0013] King, R. H. 2000. Factors that influence milk production in well-fed sows. J. Anim. Sci. 78:19–25. doi: 10.2527/2000.78suppl_319x 10947084

[CIT0014] Labarca, C., and K. Paigen. 1980. A simple, rapid, and sensitive DNA assay procedure. Anal. Biochem. 102:344–352. doi: 10.1016/0003-2697(80)90165-7 6158890

[CIT0015] Manjarin, R., N. L. Trottier, P. S. Weber, J. S. Liesman, N. P. Taylor, and J. P. Steibel. 2011. A simple analytical and experimental procedure for selection of reference genes for reverse-transcription quantitative PCR normalization data. J. Dairy Sci. 94:4950–4961. doi: 10.3168/jds.2011-4147 21943746

[CIT0016] Plante, P. -A., J. -P. Laforest, and C. Farmer. 2011. Effect of supplementing the diet of lactating sows with NuPro® on their performances and that of their piglets. Can. J. Anim. Sci. 91:295–300. doi: 10.4141/cjas2010-008

[CIT0017] Sorensen, M. T., K. Sejrsen, and S. Purup. 2002. Mammary gland development in gilts. Livest. Prod. Sci. 75:143–148. doi: 10.1016/s0301-6226(01)00310-4

[CIT0018] VanKlompenberg, M. K., R. Manjarin, J. F. Trott, H. F. McMicking, and R. C. Hovey. 2013. Late gestational hyperprolactinemia accelerates mammary epithelial cell differentiation that leads to increased milk yield. J. Anim. Sci. 91:1102–1111. doi: 10.2527/jas.2012-5903 23296835

[CIT0019] Volkin, E., and W. E. Cohn. 1954. Estimation of nucleic acids. Methods Biochem. Anal. 1:287–305. doi: 10.1002/9780470110171.ch11 13193533

[CIT0020] Weldon, W. C., A. J. Thulin, O. A. MacDougald, L. J. Johnston, E. R. Miller, and H. A. Tucker. 1991. Effects of increased dietary energy and protein during late gestation on mammary development in gilts. J. Anim. Sci. 69:194–200. doi: 10.2527/1991.691194x 2005013

